# Correlative Magnetic Resonance Imaging and Histopathology in Small Ruminant Listeria Rhombencephalitis

**DOI:** 10.3389/fneur.2020.518697

**Published:** 2020-12-14

**Authors:** Christina Precht, Peter Vermathen, Diana Henke, Anne Staudacher, Josiane Lauper, Torsten Seuberlich, Anna Oevermann, Daniela Schweizer-Gorgas

**Affiliations:** ^1^Clinical Radiology, Department of Clinical Veterinary Medicine, Vetsuisse-Faculty, University of Bern, Bern, Switzerland; ^2^Magnetic Resonance Spectroscopy and Methodology, Department of Biomedical Research, Faculty of Medicine, University of Bern, Bern, Switzerland; ^3^Division of Neurology, Department of Clinical Veterinary Medicine, Vetsuisse-Faculty, University of Bern, Bern, Switzerland; ^4^Clinic for Ruminants, Department of Clinical Veterinary Medicine, Vetsuisse-Faculty, University of Bern, Bern, Switzerland; ^5^Neurocenter, Department of Clinical Research and Veterinary Public Health, Vetsuisse-Faculty, University of Bern, Bern, Switzerland

**Keywords:** contrast enhancement, goat, sheep, listeria rhombencephalitis, listeriosis, *Listeria monocytogenes*

## Abstract

**Background:** Listeria rhombencephalitis, infection of the brainstem with *Listeria monocytogenes*, occurs mainly in humans and farmed ruminants and is associated with high fatality rates. Small ruminants (goats and sheep) are a large animal model due to neuropathological similarities. The purpose of this study was to define magnetic resonance imaging (MRI) features of listeria rhombencephalitis in naturally infected small ruminants and correlate them with histopathology. Secondly, the purpose of this study was to compare the results with MRI findings reported in humans.

**Methods:** Twenty small ruminants (13 sheep and 7 goats) with listeria rhombencephalitis were prospectively enrolled and underwent *in vivo* MRI of the brain, including T2-weighted, fluid attenuation inversion recovery, and T1-weighted sequences pre- and post-contrast administration and postmortem histopathology. In MRI, lesions were characterized by location, extent, border definition, signal intensity, and contrast enhancement. In histopathology, the location, cell type, severity, and chronicity of inflammatory infiltrates and signs of vascular damage were recorded. In addition, histopathologic slides were matched to MRIs, and histopathologic and MRI features were compared.

**Results:** Asymmetric T2-hyperintense lesions in the brainstem were observed in all animals and corresponded to the location and pattern of inflammatory infiltrates in histopathology. Contrast enhancement in the brainstem was observed in 10 animals and was associated with vessel wall damage and perivascular fibrin accumulation in 8 of 10 animals. MRI underestimated the extension into rostral brain parts and the involvement of trigeminal ganglia and meninges.

**Conclusion:** Asymmetric T2-hyperintense lesions in the brainstem with or without contrast enhancement can be established as criteria for the diagnosis of listeria rhombencephalitis in small ruminants. Brainstem lesions were similar to human listeria rhombencephalitis in terms of signal intensity and location. Different from humans, contrast enhancement was a rare finding, and abscessation was not observed.

## Introduction

Listeriosis is an important foodborne infection with the facultative intracellular bacterium *Listeria monocytogenes* (LM) occurring mainly in humans and farmed ruminants (1, 2). LM infection may involve various organ systems causing a corresponding variability of clinical syndromes, including febrile gastroenteritis, septicemia, central nervous system infection (neurolisteriosis), abortion, among others. Of those, neurolisteriosis is the fatal form and one of the leading causes of meningoencephalitis worldwide (1, 3, 4). In humans, neurolisteriosis generally develops as diffuse meningitis or meningoencephalitis, whereas between 1 and 24% of patients suffer from brainstem involvement or rhombencephalitis (5–10). Rhombencephalitis is a term interchangeably used with brainstem encephalitis and defined as the isolated infection of the brainstem and optionally the cerebellum. It was first described in humans by Eck in 1957 (11). Listeria rhombencephalitis is often not clearly distinguished from listeria meningoencephalitis and meningitis and is therefore probably underdiagnosed (7, 12).

In ruminants (cattle, sheep, and goats), the phenotype of neurolisteriosis is very consistently presenting as rhombencephalitis (7, 13). Additionally, it belongs to the most common central nervous system disorders in ruminants (14, 15). Similar to the disease in humans, the mortality rate in ruminants is high, and treatment with antibiotics in the early stage of the disease is particularly important (1, 4, 6).

Listeria rhombencephalitis may be difficult to diagnose both in humans and ruminants, as the brainstem location may not be recognized readily. Markers of inflammation in the blood and cerebrospinal fluid may be normal or only reveal mild and non-specific findings (16). Bacterial culture is time-consuming and might turn out negative, hence delaying diagnosis and initiation of appropriate treatment with antimicrobials potentially causing adverse outcomes (9, 16). Therefore, magnetic resonance imaging (MRI) could be a potentially promising tool for the rapid diagnosis of neurolisteriosis. Although recently a study on imaging of human neurolisteriosis was published (10), systematic investigation of MRI features of human rhombencephalitis is limited (6, 9, 17–20). Sporadic case studies have shown that although not pathognomonic for Listeria rhombencephalitis, MRI features in combination with clinical symptoms and laboratory findings may help to narrow down the differential diagnosis in human patients (21).

Listeria rhombencephalitis of small ruminants (sheep and goats) is very similar to that in humans with regard to neuropathology and, therefore, can be regarded as a natural disease model (2, 22). MRI imaging features of only two Listeria rhombencephalitis cases are reported in ruminants (23). This study aimed to define MRI features of listeria rhombencephalitis in naturally infected small ruminants by correlating them with histopathology to refine diagnostic criteria for Listeria rhombencephalitis. Eventually, MRI features of ruminant rhombencephalitis were compared with MRI findings reported in humans.

## Materials and Methods

### Selection of Animals

The Department of Clinical Veterinary Medicine prospectively enrolled small ruminants with the suspicion of neurolisteriosis based on neurological signs located to the brainstem and/or forebrain. All animals were examined by MRI and killed due to the severity of clinical signs and poor prognosis with the consent of the owners. Approval for the animal study was obtained from the Cantonal Ethical Committee (Swiss Veterinary Service, Office of Agriculture and Nature, approval number BE 56/10). Animals were included in the MRI analysis after diagnosis of rhombencephalitis by histopathology and further confirmation by detection of LM with immunohistochemistry and/or bacterial isolation from the medulla oblongata (24, 25). From cases in which LM was isolated (*n* = 14), information on genotype {phylogenetic lineage, sequence type [clonal complex (CC)]} based on multilocus-sequence typing was retrieved from a former study (26). Animals suffering from a neurologic disease other than neurolisteriosis were excluded based on histopathologic examination.

### Magnetic Resonance Imaging

*In vivo* MRI of the brain was performed under general anesthesia in sternal recumbency in a whole-body MRI system (Hitachi Airis II, 0.3T, Hitachi Medical Systems, Düsseldorf, Germany, and Philips Panorama HFO, or a 1.0T, Philips Medical Systems Nederland B.V., Best, Netherlands) using a quadrature head coil. Fast spin-echo (SE) T2-weighted (T2w) sequences in a transverse and sagittal plane, a fluid attenuation inversion recovery (FLAIR) sequence in a dorsal plane, a T1-weighted (T1w) three-dimensional gradient-echo sequence, and SE T1w sequences in a transverse plane before and after (directly after injection and 15 min delayed) intravenous administration of gadodiamide (0.5 mmol/ml, Omniscan, GE Healthcare, Germany) at a dose of 0.15 mmol/kg were performed. In addition, a T2^*^-weighted gradient echo sequence in the transverse plane was performed in the animals scanned with the 1.0T system. Further details of imaging parameters are available in Supplementary Table 1. Defined brain regions on the MRI were reviewed in consensus by two veterinary radiologists (DS, CP) who were blinded to neuropathological but not to clinical information. The presence of signal abnormality of the brain parenchyma in T2w, FLAIR, and T1w imaging (hyperintense, hypointense, or isointense relative to gray matter) was recorded. Lesions were characterized in terms of extent, location [brainstem subdivided into caudal (obex to the facial nerve) and rostral (facial nerve to pons) brainstem, midbrain, thalamus, striatal body, and corona radiata], border definition, signal intensity in the different sequences, signal void in the T2^*^-weighted sequence, and contrast enhancement. Evaluating the left and right side individually, lesions were graded as mild if affecting <30%, as moderate if affecting 30–60%, and as severe if affecting >60% of the parenchyma. Meninges and trigeminal ganglions were evaluated for signs of inflammation (abnormal signal intensity, thickening, and/or contrast enhancement in more than one contiguous slice).

### Histopathology

All animals were euthanized after the MRI examination, and the brain was removed. Brains were immersion-fixed in 4% neutral-buffered formaldehyde, and standard sections from the trigeminal ganglion, brainstem, cerebellum, midbrain, thalamus, striatal body, corona radiata, and cortex were embedded in paraffin, sectioned at 5 μm, and stained with hematoxylin and eosin before histopathological examination. The slides were evaluated by a veterinary neuropathologist (AO), who was blinded to imaging information but not blinded to clinical information. During histopathological analysis, topography, type (neutrophils, macrophages, lymphocytes, and plasma cells), severity, and chronicity of inflammatory infiltrates, edema, necrosis, and signs of vascular damage were recorded. Chronicity was evaluated based on the proportion of leukocyte populations within microabscesses, the cardinal histopathological lesion of neurolisteriosis (2, 27). Acute neurolisteriosis was diagnosed in cases in which neutrophils were the predominant leukocyte population within all microabscesses and perivascular cuffs with lymphocytes and macrophages were mild. Subacute neurolisteriosis cases were characterized by the presence of microabscesses with similar proportions of neutrophils and macrophages and prominent perivascular cuffs. Chronic neurolisteriosis was diagnosed in cases in which microabscesses contained macrophages predominantly and only a few neutrophils and perivascular cuffs were prominent.

### Comparison of Magnetic Resonance Imaging and Histopathology

In addition, MRI features were compared with histopathological features by simultaneous evaluation of the corresponding MRI and histopathologic slide by a radiologist (CP) and a pathologist (AO).

## Results

### Animals

Twenty-seven small ruminants (eighteen sheep and nine goats) were identified as potential participants in our study based on suspicion of neurolisterosis. Seven animals (five sheep and two goats) were excluded, as they were suffering from another neurologic disease based on histopathologic examination [clostricial encephalomalacia (2), polioencephalomalacia (1), abscess within the basioccipital bone (1), brain abscess (1), viral cerebellitis (1), and larva migrans (1)]. Twenty animals (13 sheep and 7 goats) were enrolled after confirmation of neurolisteriosis based on histopathological examination and detection of LM by bacteriological culture and/or immunohistochemistry of brain tissue. All animals were female. Further data concerning clinical data of the animals and genotypic data of bacteria are contained in Table 1. Six animals (4 sheep and 2 goats) were examined using the 0.3T and 14 animals (9 sheep and 5 goats) using the 1.0T MRI system.

**Table 1:** Clinical data, histopathology, genotypic data of bacteria, and magnetic field strength in cases with confirmed neurolisterosis.

**Parameter**		**Number of sheep**	**Number of goats**	**Total number of animals**
Age	Juvenile (<1 year)	2	1	3
	Adult (>1 year)	2 (unknown age) 9 (mean 3.7 years, range 1–6 years)	1 (unknown age) 5 (mean 3.2 years, range 1–8 years)	17
Onset of clinical signs prior to presentation	≤ 1 week	3	3	6
	1–3 weeks	2	0	2
	≥3 weeks	1	0	1
	Unknown	7	4	11
Clinical signs on presentation	Obtundity and recumbency	10	4	14
	Circling	3	3	6
	Cranial nerve deficits	8	6	14
Histopathology	Subacute	5	5	10
	Chronic	8	2	10
Phylogenetic lineage of *Listeria monocytogenes, n* = 14	I	8	3	11
	II	1	2	3
Sequence type (CC), *n* = 14	1	1	3	4
	4	7	0	7
	37	0	1	1
	394	0	1	1
	403	1	0	1
Magnetic field strength (Tesla)	0.3	4	2	6
	1.0	9	5	14

### Magnetic Resonance Imaging

**Figure 1:**
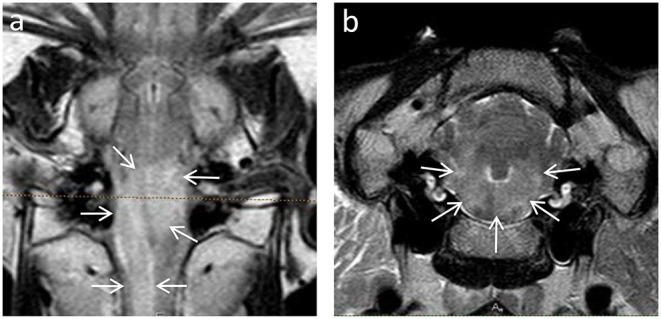
Asymmetric hyperintense signal intensity in the brainstem of animals with Listeria rhombencephalitis identified by dorsal FLAIR **(a)** and transverse T2-weighted **(b)** images. Note the asymmetric hyperintense signal (arrows) affecting most of the brainstem.

MRI features were highly consistent between animals and between bacterial genotypes. In MRI, all animals demonstrated asymmetrically increased signal intensity in the brainstem on FLAIR and T2w images (Figure 1). In T1w images, lesions were isointense in nine animals (seven sheep and two goats), iso- to hypointense in four animals (three sheep and one goat), and hypointense in seven animals (three sheep and four goats). No signal void was detected in any animal in which a T2^*^ sequence was available (14 animals, 9 sheep, and 5 goats).

**Figure 2:**
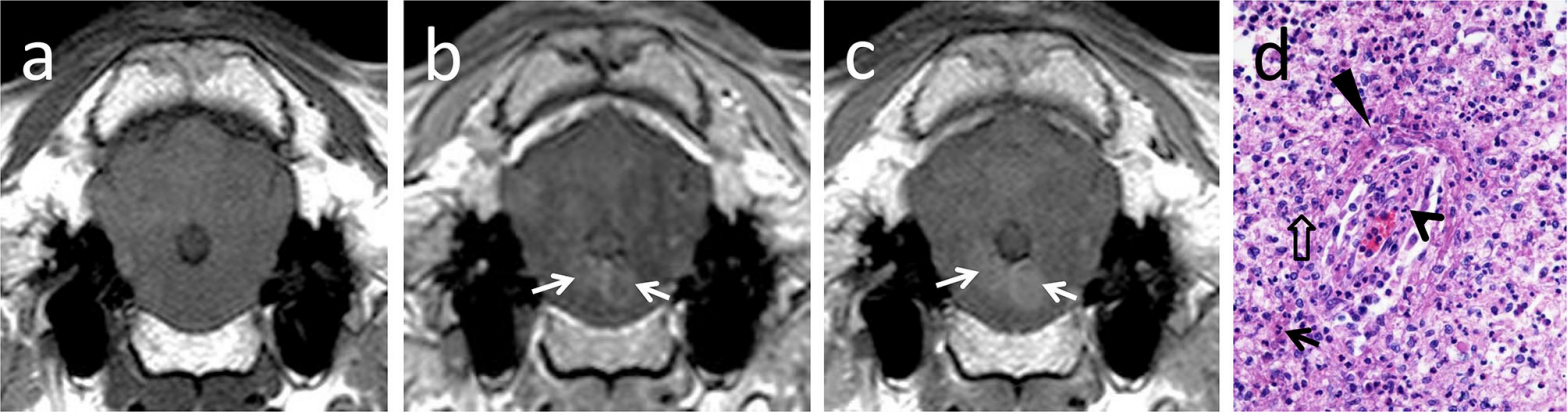
T1-isointense lesions bilaterally in the brainstem **(a)**, showing early ring-like contrast enhancement (**b**, arrows) and central diffusion of contrast with time (**c**, arrows). In histopathology **(d)**, contrast-enhancing areas corresponded to vascular damage in brainstem areas heavily infiltrated with neutrophils (arrow) and macrophages (open arrow). Vascular damage and increased permeability are indicated by infiltration of the vascular wall with neutrophils (open arrowhead), endothelial cell hypertrophy, and perivascular fibrin accumulation (arrowhead).

Parenchymal contrast enhancement was observed in the brainstem in 8 out of 14 animals examined using the 1.0T system (three sheep and five goats). Enhancement was multifocal in six animals (one sheep and five goats) and focal in two animals (both sheep) (Figure 2). Contrast enhancement was noted in none of the six animals scanned with the 0.3T system.

Ring-like enhancement was detected in two animals (one sheep and one goat). Additionally, in two animals (both sheep), focal questionable contrast enhancement was observed in a late T1w sequence 15 min after contrast administration.

### Histopathology

**Figure 3:**
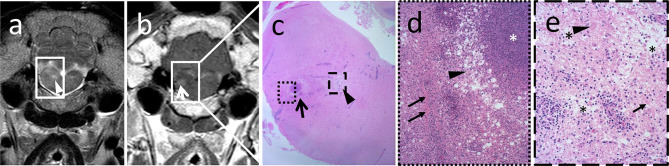
Magnetic resonance images correlate with location and grade of inflammatory infiltrates in histopathology. Hyperintense focus (**a**, arrowhead) in the transverse T2-weighted image **(a)** corresponds to focal marked edema (**c**, arrowhead) in histopathology, whereas the area of contrast enhancement (**b**, arrow) in the transverse T1-weighted sequence **(b)** corresponds to perivascular fibrin accumulation adjacent to a microabscess (**c**, arrow). In the higher magnification **(d)**, a large suppurative area with central dense aggregates of degenerated neutrophils (**d**, asterisk) surrounded by an edematous rim (**d**, arrowhead) and an extensive infiltrate with viable neutrophils and macrophages is observed. In the latter, vessels are surrounded by aggregated fibrin (**d**, arrows). Edematous area outlined in **(c)** shows pallor and disruption of the neuroparenchyma (**e**, asterisks) in the higher magnification due to strong edema and axonal (**e**, arrow) and neuronal (**e**, arrowhead) necrosis. Inflammatory infiltrates are milder in this area, and vascular damage as in the contrast-enhancing area is not observed.

**Figure 4:**
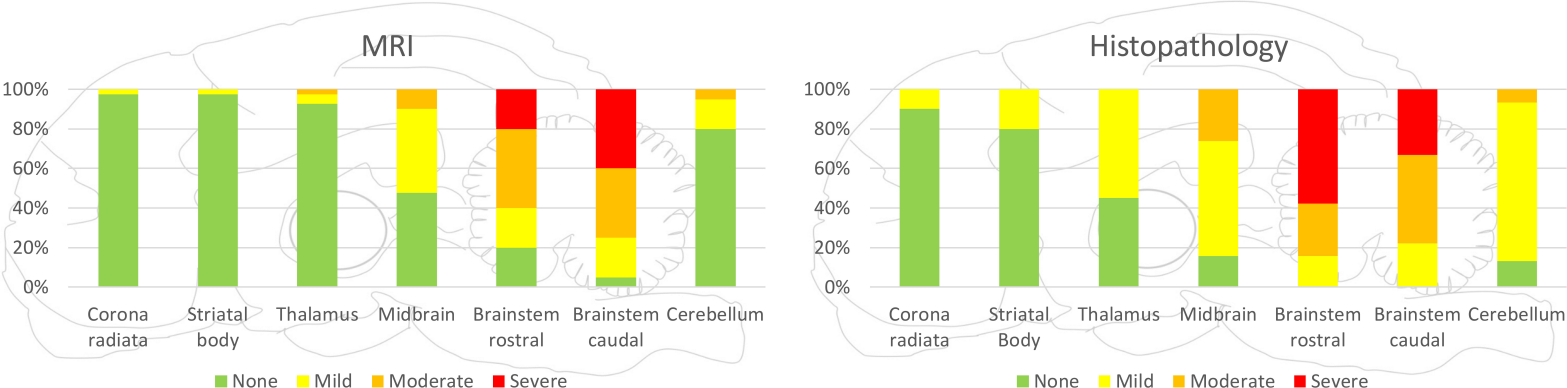
Severity of lesions in all small ruminants detected by MRI and histopathology in the various brain areas. Brainstem was most commonly and most severely affected in MRI and histopathology. Incidence and severity were decreasing from caudal to rostral brain areas, both in MRI and histopathology, whereas in histopathology, more regions were observed to be affected than in MRI.

**Figure 5:**
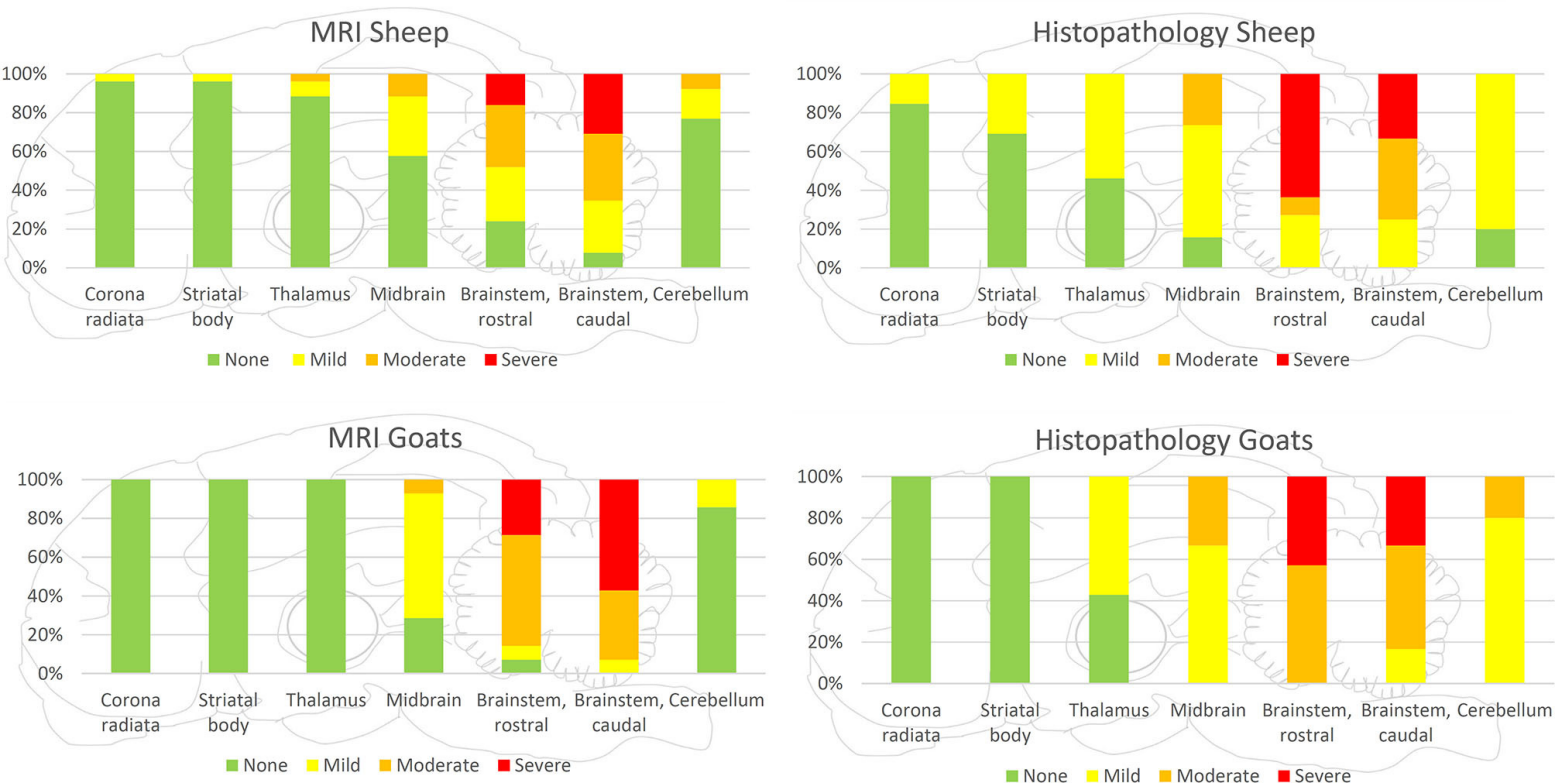
Severity of lesions detected by MRI and histopathology separately presented for goats and sheep. Both in histopathology and MRI, lesions were more severe in the brainstem and midbrain of goats compared with sheep, but the rostral extension of lesions was more commonly observed in sheep.

Similar to MRI, histopathological lesions of listeria rhombencephalitis were strongly consistent between animals and bacterial genotypes. In all animals, lesions consisted of prominent lymphohistiocytic perivascular cuffs associated with the presence of pathognomonic microabscesses containing various proportions of neutrophils and macrophages. Ten animals suffered from subacute rhombencephalitis (five sheep and five goats) with similar proportions of neutrophils and macrophages within microabscesses and 10 animals from chronic rhombencephalitis (eight sheep and two goats) with a marked predominance of macrophages within microabscesses, respectively. Additionally, all animals exhibited varying levels of parenchymal edema, necrosis, and meningitis. In 10 animals with extensive inflammatory infiltrates characterized by coalescing microabscesses, vessels within these areas were involved in the inflammatory process exhibiting vasculitis, protein leakage, and fibrin exudation (Figure 3; five sheep and five goats). Inflammatory lesions were targeted clearly on the brainstem but frequently extended to the midbrain and thalamus (Figures 4, 5, Supplementary Table 2).

### Comparison of Magnetic Resonance Imaging and Histopathology

In both MRI and histopathology, the brainstem was the most severely affected region. In MRI, the distribution pattern of lesions ranged from patchy and unilaterally to diffusely affecting the entire cross-sectional area of the brainstem with an asymmetric distribution. In histologically analyzed brain sections, all animals showed inflammatory changes on both sides of the brainstem. However, severity was frequently asymmetric as well in histopathology, and areas of signal change in MRI clearly corresponded to inflammatory changes in histopathology (Figure 3). Data on lesion distribution and the correlation between MRI and histopathology are presented in Figures 4, 5; numerical data are given in Supplementary Table 2.

**Figure 6:**
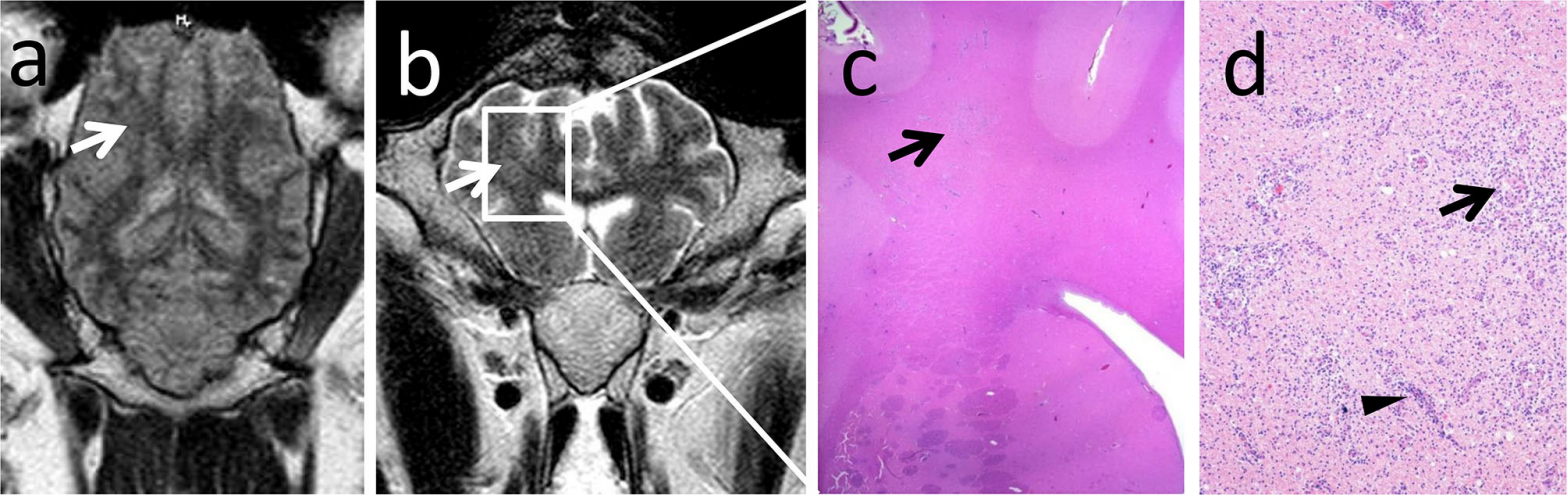
MRI rarely detected lesion extension rostrally to the midbrain. Note the unilateral focal hyperintense signal in the corona radiata in the dorsal FLAIR (**a**, arrow) and transverse T2-weighted (**b**, arrow) sequences corresponding to a chronic inflammatory infiltrate in histopathology (**c**, arrow). Chronic inflammatory infiltrate consists of coalescing areas of macrophage infiltrations (**d**, arrow) and lymphohistiocytic perivascular cuffs (**d**, arrowhead) in the gliotic white matter.

In the cerebellum, midbrain, and more rostral areas, incidence and severity of lesions decreased with increasing distance from the brainstem in both MRI and histopathology. Lesions detected by MRI corresponded to the locations of histopathologically detected inflammatory infiltrates (Figure 6). Conversely, histopathologically detectable lesions were not always visible on MRI. Data on lesion distribution are presented in Figures 4, 5; numerical data are given in Supplementary Table 2.

Contrast enhancement was observed commonly in subacute and rarely in chronic cases of Listeria rhombencephalitis. Out of the 10 animals with subacute listeriosis, seven animals (two sheep and five goats) showed clear and one animal (one sheep) questionable contrast enhancement. Only two animals (both sheep) with subacute listeriosis did not show contrast enhancement. Out of the 10 animals with chronic listeriosis, only one animal (one sheep) showed clear and one animal (one sheep) questionable contrast enhancement, but eight animals (six sheep and two goats) did not show contrast enhancement.

In most animals with contrast enhancement, perivascular fibrin accumulation indicating vascular damage was observed in histopathology in the brain parenchyma at sites corresponding to the contrast-enhancing region in MRI (Figure 2): Seven of the eight animals (two out of three sheep and all five goats, respectively) and one of the two animals (one out of two sheep) with questionable contrast enhancement had lesions of vascular damage in histopathology. In contrast, perivascular fibrin accumulation was observed in histopathology only in two animals without contrast enhancement (two sheep, both scanned with the 1.0T system).

Trigeminal ganglion neuritis was more commonly detected by histopathology compared with MRI. MRI analysis did not detect abnormality in 18 histopathologically abnormal trigeminal ganglia (false negative) and judged three trigeminal ganglia as questionably abnormal, which were normal in histopathology (false positive). Only two trigeminal ganglia were evaluated as abnormal and 11 as normal in both MRI and histopathology.

Meningitis was detected histopathologically in all animals and frequently mild. In contrast, focal meningeal contrast enhancement was detected in five animals only (four sheep and one goat).

## Discussion

The main characteristic MRI feature identified in goats and sheep with histopathologically confirmed Listeria rhombencephalitis was the presence of asymmetric T2-hyperintense lesions in the brainstem with or without contrast enhancement, independently of the host species and the genotype of LM.

### Comparison of Magnetic Resonance Imaging and Histopathology in Small Ruminants

When comparing MRI and histopathologic findings, both methods agreed on the brainstem being the most commonly and severely affected region of the brain. Comparing histopathology slides and MRIs of the brainstem, the pattern and distribution of the lesions corresponded to each other. The T2-hyperintense regions reflect a high-water lesion due to inflammatory changes characterized by perivascular mononuclear cuffings, intraparenchymal microabscesses, edema, focal areas of necrosis, and gliosis. These histopathological features are very similar in humans and ruminants (2, 22). Contrast enhancement in T1w sequences with a focal, multifocal, or ring-like pattern in the brainstem was linked to perivascular fibrin accumulation indicating breakdown of the blood–brain barrier, which was most commonly observed in cases with subacute inflammation (mainly goats), but rarely in cases with chronic features of inflammation (mainly sheep). Ring-like-enhancing lesions corresponded to an area of coalescing microabscesses in histopathology. These lesions lacked other imaging and histopathological characteristics of abscesses.

Histopathologically, lesions were most severe in the brainstem but were not restricted to the medulla and pons in most animals. Lesions were consistently found rostrally to the mesencephalon, including diencephalon and telencephalon, with decreasing severity from the lower brainstem toward the cerebrum and cerebellum, in line with previous reports in ruminants (2). This gradient was frequently associated with an increased relative proportion of neutrophilic microabscesses vs. microabscesses with predominantly macrophages in a rostral direction, indicating caudorostral spread (2). Although brainstem lesions were more severe in goats than in sheep, inflammation extended more extensively into rostral brain areas of sheep. The wider spread of lesions in sheep was associated with a higher incidence of histopathological features of chronicity, potentially suggesting that a delayed course of infection facilitates caudorostral spread. MRI underestimated lesions in their extension, most likely because the severity of tissue damage in more rostral brain regions and cerebellum was below the detection threshold.

Similarly, histopathologically confirmed trigeminal ganglion neuritis and meningitis were underestimated by MRI in our study. The majority of trigeminal ganglia had inflammatory infiltrates but frequently did not show any MRI detectable abnormalities. Although clinical and histopathological involvement of nuclei and tracts of cranial nerves is commonly reported in humans and ruminants as a cardinal feature of brainstem encephalitis (6, 22), detection of trigeminal nerve involvement distal to the emergence from the brainstem by MRI is described in few case reports only (20, 28). This may be explained by either involvement of the intraparenchymal part (nuclei and tracts) of the cranial nerves but not the cranial nerves distal to their emergence from the brainstem or by the difficulty to image cranial nerves due to their small size. In human Listeria rhombencephalitis, clinical signs of meningitis may be present in only 50% of cases, whereas laboratory findings, including pleocytosis and elevated proteins in the cerebrospinal fluid, are more frequently occurring in 78 and 85% of patients, respectively (6). Similar to the small ruminants in our study, MRI signs of meningitis are uncommon in human Listeria rhombencephalitis cases, whereas they are frequently detected in human Listeria meningoencephalitis (10). This is not surprising, as approximately half of the imaging studies of acute meningitis show no specific imaging abnormalities (29). We cannot totally exclude the contribution of low field strength to the low sensitivity in the detection of parenchymal, meningeal, and trigeminal lesions in 6 out of 20 animals. However, as most animals (14 out of 20) were scanned in a high field magnet (1.0T), we consider this to be a minor factor.

### Comparison of Magnetic Resonance Imaging Features in Small Ruminants and Humans

The limited number of case reports and series of Listeria brainstem encephalitis in humans, including MRI, describes T2-hyperintense lesions in the brainstem similar to the lesions that we observed in the small ruminants (6, 10, 18, 20, 30–33). Similar to our findings in small ruminants, the involvement of the mesencephalon and supratentorial brain structures on MRI is rare in human Listeria brainstem encephalitis (20, 34). In the few cases in which histopathologic examination is also available, MRI and histopathological lesions are typically restricted to the medulla and pons and rarely extend rostrally (6, 22).

However, in almost two-thirds of human Listeria rhombencephalitis, T1-contrast enhancement is reported (10, 18, 20, 22, 32). In ~50% of contrast-enhancing cases, ring-enhancing single or multiple abscesses were observed in the brainstem, cerebellum, or mesencephalon (10, 20, 32, 34). We did observe small ring-enhancing lesions that correlated with the presence of coalescing microabscesses in histopathology. However, we did not observe abscesses in the small ruminants, neither with MRI nor in histopathology. Furthermore, in some cases of human Listeria rhombencephalitis, hydrocephalus was present (20), which we did not find in any of the small ruminants. The reason for the more commonly reported contrast enhancement and development of abscesses and hydrocephalus in humans compared with small ruminants remains speculative and may be a consequence of limited case number and reporting bias. Possible explanations to consider are the time point of imaging in the disease and different pathogenesis and immune response in humans and small ruminants. Potentially, involved strains may have an impact on the type of lesions observed in MRI and histopathology. However, data regarding the association of the type of neurolisteriosis with LM sequence type and CC are lacking. Consistent with former studies in ruminants, most of the ruminants with rhombencephalitis in this study were infected with isolates from CC1 or CC4 (26), whereas in humans, the range of CCs associated with neurolisteriosis is much larger (35). Future studies need to address the potential association of genotype with the phenotype of neurolisteriosis. The more frequent contrast enhancement in human patients may indicate that neurolisteriosis is more commonly associated with severe vascular damage as compared with ruminants. In line with this assumption, hemorrhage indicating vascular damage was not observed in our study but frequently observed in a systematic imaging study of human neurolisteriosis (10).

### Evaluation of Small Ruminants as a Disease Model From the Imaging Standpoint

At present, there is a lack of agreement on an optimal animal model for human neurolisteriosis due to concerns about physiological differences among animal species (36). Ideally, an animal model should closely resemble the respective infection process in humans, mirror the infection endpoint of interest, and include enough animals to be statistically relevant in biological variability and minimizing uncertainty (37). The mouse has been used as a model to study the pathogenesis of systemic listeriosis (38). However, as mice are not naturally susceptible to LM, the routes frequently used for experimental infection do not reflect those of natural infection. Thus, small ruminants, which show similar characteristics to human infection, especially in terms of histopathologic criteria, have been suggested as a relevant model to study the pathogenesis of neurolisteriosis (2, 22). If we evaluate the suitability of small ruminants as an animal model from the imaging standpoint, we should appreciate that small ruminants suffer from the rhombencephalitis type of neurolisteriosis and not the meningoencephalitis type. Our data showed that there are important similarities of MRI features between human and ruminant rhombencephalitis, especially the asymmetric T2-hyperintensities localized within the brainstem. However, there are also important differences, such as the frequency of contrast enhancement and the occurrence of abscesses and hydrocephalus. Nevertheless, we think that our study supports the use of small ruminants as a model for Listeria rhombencephalitis in humans. Although the animals studied originated from different regions of Switzerland and were naturally infected, they showed very consistent MRI features, indicating uniform development of pathophysiologic processes, favoring the use as an animal model.

### Magnetic Resonance Imaging as a Diagnostic Tool

The previous findings of two small ruminants with neurolisteriosis were confirmed in this systematic study (23). We cannot claim that the MRI features of Listeria rhombencephalitis that we reported in small ruminants are specific, as we did not systematically compare MRI features of Listeria rhombencephalitis with other neurologic diseases due to lack of cases. Imaging features of other common neurologic diseases have been reported as single cases only (23). However, due to a lack of differentials with similar brainstem predilection in small ruminants (15, 39), it seems likely that asymmetric T2-hyperintense lesions in the brainstem with or without contrast enhancement can be established as criteria for the diagnosis of Listeria rhombencephalitis in these species. Due to financial restrictions, it likely will not be used as a routine diagnostic tool in veterinary practice. However, in case of large outbreaks where the intention is not the diagnosis of individual disease but the protection of the remaining flock, MRI may be a valuable tool to come to a quick presumptive diagnosis. In humans, several differential diagnoses with brainstem predilection have to be considered, for example, demyelinating (e.g., multiple sclerosis, acute disseminated encephalomyelitis), autoimmune/inflammatory (Behcet's disease, paraneoplastic), infectious (herpesvirus, tuberculosis), and very rarely neoplastic diseases (e.g., lymphoma) (6, 9, 40, 41). Nevertheless, MRI features combined with clinical symptoms and laboratory findings may be clearly indicative of Listeria rhombencephalitis (21).

### Limitations of the Study

Limitations of our study are the rather low number of animals and the unblinded evaluation by the observers. Listeria brainstem encephalitis is a rare disease with severe clinical symptoms, which hampers the recruitment of naturally infected animals into an *in vivo* study. However, in relation to the number of human case reports, including MRI description, our case number seems to be reasonable to undertake a comparison between the MRI features in both species. In addition, the low field strength used in 6 out of 20 animals may have influenced the sensitivity to detect subtle morphological changes.

### Conclusion

In conclusion, asymmetric T2-hyperintense lesions in the brainstem were the main MRI feature in small ruminants with Listeria brainstem encephalitis similar to human cases and matched with the location and pattern of inflammatory infiltrates in histopathology. Contrast enhancement in the brainstem was an inconsistent finding associated with perivascular fibrin accumulation in subacute cases but not with abscessation as reported in humans. MRI underestimated the extension into rostral brain parts and the involvement of trigeminal ganglia and meninges. We could demonstrate important similarities and some differences between MRI features of Listeria brainstem encephalitis in small ruminants and humans.

## Data Availability Statement

The datasets generated for this study are available on request to the corresponding author.

## Ethics Statement

The animal study was reviewed and approved by Animal Welfare and animal testing comission, Office of Agriculture and Nature, Veterinary Service, Canton Bern, Bern, Switzerland. Written informed consent was obtained from the owners for the participation of their animals in this study.

## Author Contributions

CP, DS-G, AO, TS, and PV contributed conception and design of the study. CP, DS-G, JL, AS, and DH were involved in the acquisition and organization of clinical and/or MRI data. CP and DS-G evaluated MRI data. AO evaluated histopathologic data. CP wrote the first draft of the manuscript. All authors contributed to manuscript revision and read and approved the submitted version.

## Conflict of Interest

The authors declare that the research was conducted in the absence of any commercial or financial relationships that could be construed as a potential conflict of interest.
