# Supplementary material for: Correlative Magnetic Resonance Imaging and Histopathology in Small Ruminant Listeria Rhombencephalitis
Source: Front Neurol. 2020 Dec 14;11:518697. doi: 10.3389/fneur.2020.518697 (PMC7773005; doi:10.3389/fneur.2020.518697)
Supplement: Supplementary file 1 [file Data_Sheet_1.docx]

Supplementary Material

# Supplementary tables

**Supplementary table 1** Imaging parameters for fast spin echo (SE) T2-weighted (T2w), Fluid Attenuation Inversion Recovery (FLAIR), T2*-weighted (T2*w), SE T1-weighted (T1w), and gradient echo (GE) 3D T1-w sequences

|  |  | **1.0 Tesla system** | **0.3 Tesla system** |
| --- | --- | --- | --- |
| **T2w sag** | **TR** | 3000 | 4000 |
|  | **TE** | 100 | 120 |
|  | **Slice thickness** | 3.5 | 2.5 |
|  | **gap** | 0.5 | 0.5 |
| **T2w tra** | **TR** | 5920 | 6620 |
|  | **TE** | 100 | 125 |
|  | **Slice thickness** | 3.5 | 3.5 |
|  | **gap** | 0.5 | 0.5 |
| **FLAIR dor** | **TR** | 11000 | 8031 |
|  | **TE** | 140 | 125 |
|  | **TI** | 2800 | 1900 |
|  | **Slice thickness** | 3.5 | 4 |
|  | **gap** | 1 | 0.5 |
| **T2*w tra** | **TR** | 500 | - |
|  | **TE** | 20,7 | - |
|  | **Flip angle** | 18 | - |
|  | **Slice thickness** | 3.5 | - |
|  | **gap** | 0.5 | - |
| **SE T1w tra** | **TR** | 400 | 520 |
|  | **TE** | 15 | 20 |
|  | **Flip angle** | 80 | 90 |
|  | **Slice thickness** | 3.5 | 3 |
|  | **gap** | 0.5 | 1 |
| **GE 3D T1w tra** | **TR** | 27 | 30 |
|  | **TE** | 7 | 12 |
|  | **Flip angle** | 30 | 30 |
|  | **Slice thickness** | 0.9 | 1 |
|  | **gap** | 0 | 0 |

Repetition time (TR) in ms, echo time (TE) in ms, inversion time (TI) in ms, flip angle in °, slice thickness and gap in mm

**Supplementary table 2** Severity of lesions detected by MRI and histopathology in goats and sheep affected by listeria rhombencephalitis

|  |  | **MRI**  subdivided into left and right side for each animal (n=40), for the cerebellum no. of animals n=20 | | | | **Histopathology**  no. of animals (n=20) | | |
| --- | --- | --- | --- | --- | --- | --- | --- | --- |
|  |  | **Total number of animals** | Number of sheep | | Number of goats | **Total number of animals** | Number of sheep | Number of goats |
| Brainstem, caudal part | Mild  Moderate  Severe  None | **8/40**  **14/40**  **16/40**  **2/40** | 7/26  9/26  8/26  2/26 | 1/14  5/14  8/14  0/14 | | **4/18**  **8/18**  **6/18**  **0/18** | 3/12  5/12  4/12  0/12 | 1/6  3/6  2/6  0/6 |
| Brainstem, rostral part | Mild  Moderate  Severe  None | **8/40**  **16/40**  **8/40**  **8 /40** | 7/26  8/26  4/26  6/26 | 1/14  8/14  4/14  1/14 | | **3/19**  **5/19**  **11/19**  **0/19** | 3/12  1/12  7/12  0/12 | 0/7  4/7  3/7  0/7 |
| Midbrain | Mild  Moderate  Severe  None | **17/40**  **4/40**  **0/40**  **19/40** | 8/26  3/26  0/26  15/26 | 9/14  1/14  0/14  4/14 | | **11/19**  **5/19**  **0/19**  **3/19** | 7/13  3/13  0/13  3/13 | 4/6  2/6  0/6  0/6 |
| Thalamus | Mild  Moderate  Severe  None | **2/40**  **1/40**  **0/40**  **37/40** | 2/26  1/26  0/26  23/26 | 0/14  0/14  0/14  14/14 | | **11/20**  **0/20**  **0/20**  **9/20** | 7/13  0/13  0/13  6/13 | 4/7  0/7  0/7  3/7 |
| Striatal body | Mild  Moderate  Severe  None | **1/40**  **0/40**  **0/40**  **39/40** | 1/26  0/26  0/26  25/26 | 0/14  0/14  0/14  14/14 | | **4/20**  **0/20**  **0/20**  **16/20** | 4/13  0/13  0/13  9/13 | 0/7  0/7  0/7  7/7 |
| Corona radiata | Mild  Moderate  Severe  None | **1/40**  **0/40**  **0/40**  **39/40** | 1/26  0/26  0/26  25/26 | 0/14  0/14  0/14  14/14 | | **2/20**  **0/20**  **0/20**  **18/20** | 2/13  0/13  0/13  11/13 | 0/7  0/7  0/7  7/7 |
| Cerebellum | Mild  Moderate  Severe  None | **3/20**  **1/20**  **0/20**  **16/20** | 2/13  1/13  0/13  10/13 | 1/7  0/7  0/7  6/7 | | **12/15**  **1/15**  **0/15**  **2/15** | 8/10  0/10  0/10  2/10 | 4/5  1/5  0/5  0/5 |
